# Eckmaxol Isolated from *Ecklonia maxima* Attenuates Particulate-Matter-Induced Inflammation in MH-S Lung Macrophage

**DOI:** 10.3390/md20120766

**Published:** 2022-12-07

**Authors:** D. P. Nagahawatta, N. M. Liyanage, H. H. A. C. K. Jayawardhana, Thilina U. Jayawardena, Hyo-Geun Lee, Moon-Soo Heo, You-Jin Jeon

**Affiliations:** 1 Department of Marine Life Sciences, Jeju National University, Jeju 63243, Jeju Self-Governing Province, Republic of Korea; 2 Department of Chemistry, Biochemistry and Physics, Université du Québec à Trois-Rivières, Trois-Rivières, QC G8Z 4M3, Canada; 3 Marine Science Institute, Jeju National University, Jeju 63333, Jeju Self-Governing Province, Republic of Korea

**Keywords:** eckmaxol, *Ecklonia maxima*, anti-inflammation, particulate matter, lung macrophage, chronic diseases, bioactive compound

## Abstract

Airborne particulate matter (PM) originating from industrial processes is a major threat to the environment and health in East Asia. PM can cause asthma, collateral lung tissue damage, oxidative stress, allergic reactions, and inflammation. The present study was conducted to evaluate the protective effect of eckmaxol, a phlorotannin isolated from *Ecklonia maxima*, against PM-induced inflammation in MH-S macrophage cells. It was found that PM induced inflammation in MH-S lung macrophages, which was inhibited by eckmaxol treatment in a dose-dependent manner (21.0–84.12 µM). Eckmaxol attenuated the expression of cyclooxygenase-2 (COX-2) and inducible nitric oxide synthase (iNOS) in PM-induced lung macrophages. Subsequently, nitric oxide (NO), prostaglandin E-2 (PGE-2), and pro-inflammatory cytokines (IL-1β, IL-6, and TNF-α) were downregulated. PM stimulated inflammation in MH-S lung macrophages by activating Toll-like receptors (TLRs), nuclear factor-kappa B (NF-κB), and mitogen-activated protein kinase (MAPK) pathways. Eckmaxol exhibited anti-inflammatory properties by suppressing the activation of TLRs, downstream signaling of NF-κB (p50 and p65), and MAPK pathways, including c-Jun N-terminal kinase (JNK) and p38. These findings suggest that eckmaxol may offer substantial therapeutic potential in the treatment of inflammatory diseases.

## 1. Introduction

Particulate matter (PM) is a known threat under air pollution in urban areas. PM includes both organic materials, such as biological materials (endotoxins, fungal spores, and pollen), and inorganic elements, such as metals, salts, and carbonaceous materials [1]. Ultrafine PM (≥100 nm) does not sediment or flocculate easily and is retained in the atmosphere for a longer period as compared to other particles (2.5–10 µM). This results in the transport of PM with the wind [2,3]. Prolonged exposure to PM results in adverse effects on human health, particularly on normal lung function, by inducing inflammation and oxidative stress in lung cells [4,5]. The inhalation of PM depends on its penetration depth, deposition, particle size, shape, and density [6]. The World Health Organization (WHO) reported that PM exposure was responsible for more than seven billion deaths in 2012, whereas the American Cancer Society indicated that the rise in PM increased the total death rate by 7% [5,7,8].

Lungs are vital organs in the human body as they supply oxygen. Continuous PM inhalation has severe negative effects on the lungs. PM is known to be important in the development of chronic inflammatory lung diseases, such as asthma, chronic obstructive pulmonary disorder, and lung cancer [9]. PM mainly mediates oxidative stress in cells via reactive oxygen species (ROS), which are generated by free radicals contained in particle surfaces [10]. Both oxidative stress and PM itself activate redox-sensitive signaling pathways, which result in inflammatory responses [11]. Inflammatory responses are considered as the primary protective actions and involve the upregulation and activation of several important genes for signaling molecules such as cytokines (TNF-α, IL-6), chemokines (IL-8), and adhesion molecules [12,13].

Inflammatory cytokines are key signaling proteins synthesized and released by macrophages under stress conditions, and their quantity and interaction with the receptors control the activation of immune cells and subsequent signaling cascades [14,15]. This proves that macrophages and cytokine signaling play major regulatory roles in inflammatory processes in the lungs.

*Ecklonia maxima* is a brown seaweed commonly found in the South African coastal region that has high bioactivity. The leaves of *E. maxima* are commonly used as a source of alginates, animal feed, fertilizers, nutrient supplements, and medication preparations. Polysaccharides from this seaweed species and antioxidant have been found to have anti-diabetes and anti-cancer activities [16,17]. Eckmaxol is a phlorotannin isolated from *E. maxima* leaves [18]. Phlorotanins are polyphenolic compounds with a wide range of molecular weights that possess many potential health benefits [19]. They are formed by the polymerization of aromatic precursors through the acetate–malonate pathway [20]. Phlorotanins are considered to possess numerous bioactivities, such as anti-diabetic, anti-inflammatory, antioxidant, anti-bacterial, and anti-cancer activities [21,22,23,24,25]. The activity of eckmaxol against neurotoxicity, melanogenesis, and LPS-induced inflammation was previously evaluated [18,26,27]. However, to the best of our knowledge, the protective effect of eckmaxol against PM-induced lung inflammation has not been systematically investigated. Thus, the identification of Toll-like receptors (TLRs) responsible for the induction of inflammation in PM-exposed lung macrophages and their downstream signal transduction may be a possible target for the development of a therapeutic agent for the treatment of PM-induced lung inflammation. The purpose of the present study was to investigate the influence of PM on the inflammatory responses of lung macrophages and the protective effect of eckmaxol isolated from *E. maxima* against PM-induced inflammatory responses in the lungs.

## 2. Results

### 2.1. Characterization of PM and Identification Eckmaxol

**Figure 1:**
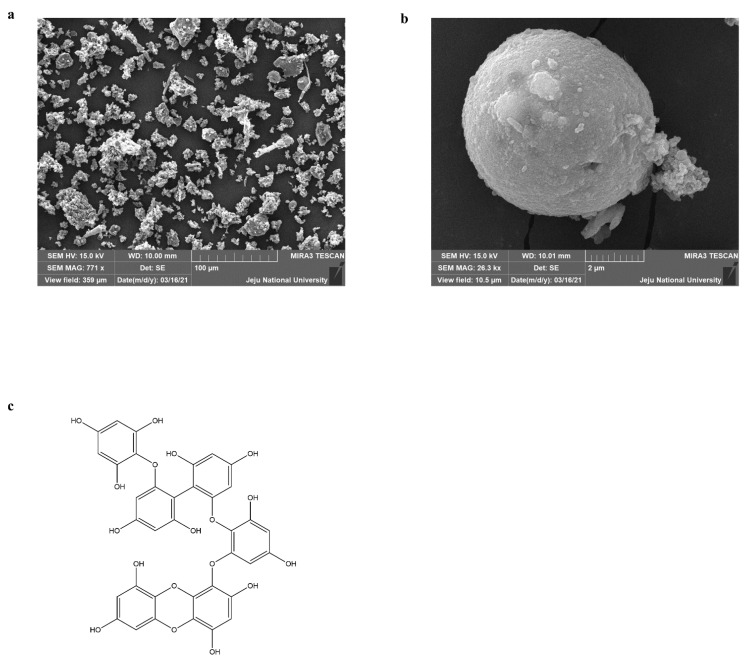
Physical parameters of particulate matter (PM) and chemical structure of eckmaxol. (**a**) Scanning electron microscopic (SEM) image, (**b**) magnified SEM image of PM particle of certified CRM No.28, National Institute for Environmental Studies (NIES), Ibaraki, Japan, and (**c**) the chemical structure of eckmaxol.

Certified reference material No.28 (Chinese PM) was used for this experiment. The detailed procedure for collecting PM via mechanical vibration and chemical characterization was described previously [28]. The particle size and distribution were evaluated by scanning electron microscopy (SEM), as shown in Figure 1a,b. This provides evidence that the majority of the particles had an average diameter of less than 5 µm. In addition, the data were provided by the National Institute for Environmental Studies (NIES), Ibaraki, Japan. The isolated compound was characterized using high-performance liquid chromatography (HPLC) and electrospray ionization (ESI). The HPLC analysis solidified that eckmaxol with high purity via analysis peak characterization (Figure S1). The recorded purity was more than 90%. ESI-MS (positive) evaluation based on the HPLC analysis confirmed the molecular weight of the eckmaxol that aligned with the previously published results [27]. The chemical structure of the eckmaxol (C_36_H_24_O_18_) was demonstrated in Figure 1c.

### 2.2. Effect of Eckmaxol on MH-S Lung Macrophages and PM-Stimulated Cell Viability and NO Production

**Figure 2:**
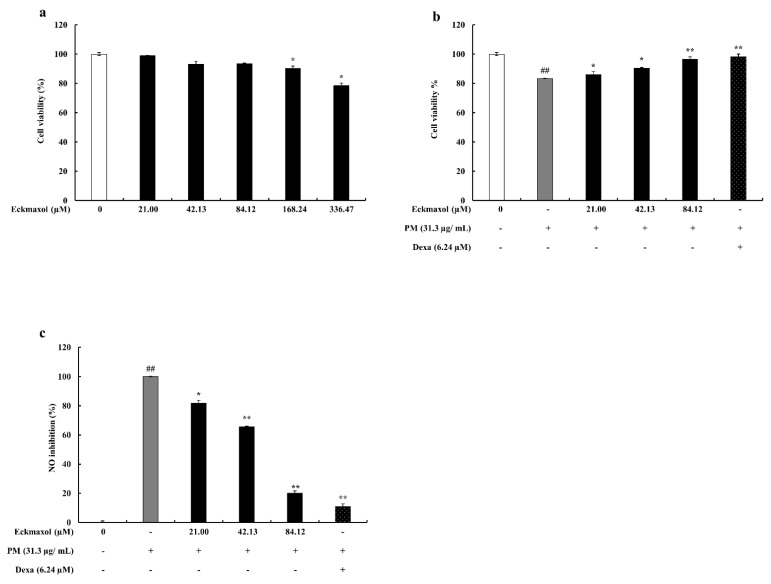
Dose-range determination and cytoprotective activity evaluation of eckmaxol on particulate matter (PM)-induced MH-S lung macrophages. (**a**) Cytotoxicity of eckmaxol, (**b**) cytoprotective effect of eckmaxol against PM, (**c**) NO production inhibition effect of eckmaxol against PM. Triplicate experiments were used to evaluate the data and the mean value is expressed with ± SD. * *p* < 0.05, ** *p* < 0.01, against PM-treated group or ## *p* < 0.01, against control (ANOVA, Duncan’s multiple range test).

According to the results, eckmaxol concentrations higher than 84.12 µM showed a cytotoxic effect on MH-S lung macrophages (Figure 2a). Therefore, eckmaxol concentrations between 21.00–84.12 µM were used for further experiments. As shown in Figure 2b, the protective effect of eckmaxol was examined against PM-stimulated MH-S lung macrophages. Cell viability was significantly affected by PM, whereas eckmaxol exhibited a significant recovery effect in a dose-dependent manner. Owing to PM, NO production was significantly upregulated, while treatment with eckmaxol significantly downregulated NO production in a dose-dependent manner (Figure 2c).

### 2.3. Preventive Effect of Eckmaxol on Prostaglandin E2 (PGE-2) and Pro-Inflammatory Cytokine Production in PM-Induced MH-S Cells

**Figure 3:**
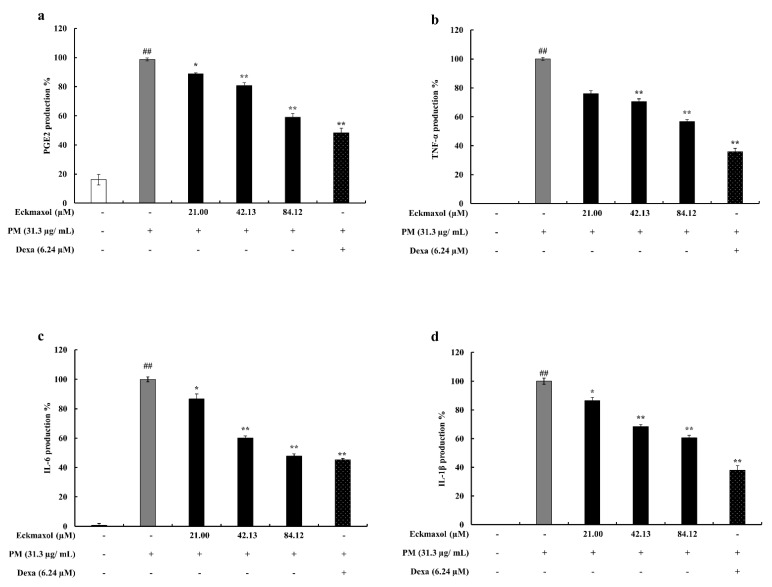
Inhibitory effect of eckmaxol against particulate matter (PM)-induced PGE-2 and pro-inflammatory cytokine produciton in MH-S lung macrophages (TNF-α, IL-6, and IL-1β) production. Inhibitory effect on (**a**) PGE-2 production, (**b**) TNF-α production, (**c**) IL-6 production, and (**d**) IL-1β production. Triplicate experiments were used to evaluate the data and the mean value is expressed with ± SD. * *p* < 0.05, ** *p* < 0.01, against PM-treated group or ## *p* < 0.01, against control (ANOVA, Duncan’s multiple range test).

Further confirmation of in vitro anti-inflammatory properties was investigated by examining the secretion levels of PGE-2 and pro-inflammatory cytokines (TNF-α, IL-6, and IL-1β) in PM-induced MH-S cells using enzyme-linked immunosorbent assay (ELISA). As shown in Figure 3, the production levels of PGE-2 and pro-inflammatory cytokines were strongly stimulated by PM, whereas the eckmaxol-treated groups showed significant and dose-dependent suppression of their production.

### 2.4. Potential of Eckmaxol to Inhibit Inducible Nitric Oxide Synthase (iNOS) and Cyclooxygenase-2 (COX-2) Gene and Protein Expression in PM Stimulated MH-S Lung Macrophages

**Figure 4:**
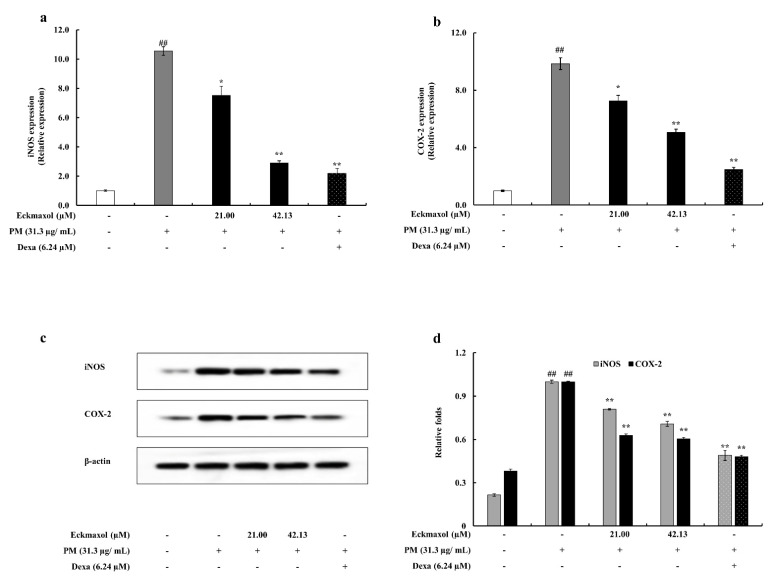
Inflammation-associated protein and gene expression of iNOS and COX-2 in particulate matter (PM)-induced MH-S lung macrophage attenuated by eckmaxol. (**a**) iNOS gene expression, (**b**) COX-2 gene expression, (**c**) iNOS and COX-2 protein expression, and (**d**) quantification of iNOS and COX-2 protein expression. Triplicate experiments were used to evaluate the data and the mean value is expressed with ± SD. * *p* < 0.05, ** *p* < 0.01, against PM-treated group or ## *p* < 0.01, against control (ANOVA, Duncan’s multiple range test). β-actin was used as the house-keeping gene. Quantitative data were analyzed using Image J software.

Protein expression of iNOS and COX-2 revealed the anti-inflammatory activity of eckmaxol via Western blotting. The results of the gene expression analysis confirmed this. According to the Western blots, the upregulated gene expression of iNOS and COX-2 was significantly decreased by eckmaxol treatment. The gene expression results for iNOS and COX-2 exhibited a similar trend (Figure 4).

### 2.5. Eckmaxol Suppresses the Pro-Inflammatory Cytokine Gene Expressions

**Figure 5:**
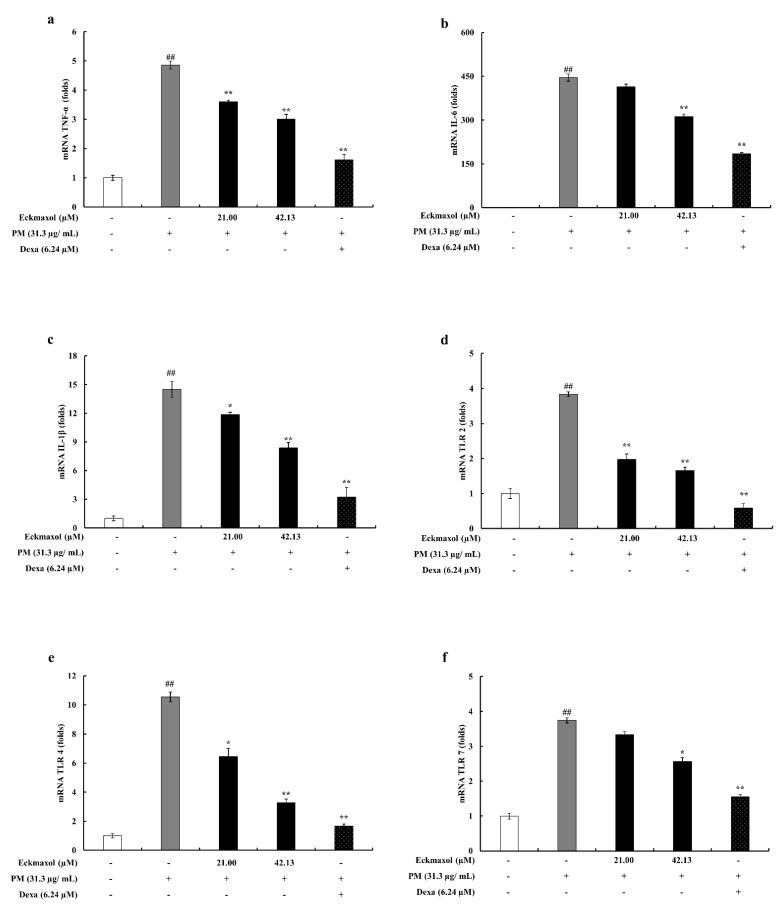
Gene expression levels evaluation in particulate matter (PM)-stimulated MH-S lung macrophages attenuated by eckmaxol. (**a**) TNF-α, (**b**) IL-6, (**c**) IL-1β, (**d**) TLR-2, (**e**) TLR-4, and (**f**) TLR-7. The mRNA expression levels were measured via RT-qPCR techniques. Triplicate experiments were used to evaluate the data and the mean value is expressed with ± SD. * *p* < 0.05, ** *p* < 0.01, against PM-treated group or ## *p* < 0.01, against control (ANOVA, Duncan’s multiple-range test).

The mRNA expression levels of selected proinflammatory cytokines (TNF-α, IL-6, and IL-1β) were evaluated to measure the inhibitory effect of eckmaxol on mRNA expression. According to the qPCR results, the gene expression levels of pro-inflammatory cytokines were significantly increased by PM exposure and the upregulated gene expression was significantly and dose-dependently decreased by eckmaxol treatment (Figure 5a–c).

### 2.6. Inhibitory Activity of Eckmaxol on the Expression of TLRs

The gene expression levels of TLRs in MH-S lung macrophages were measured using qPCR. The results revealed elevated expression of TLR-2, TLR-4, and TLR-7 in PM-stimulated MH-S lung macrophages. However, these inclined expressions were significantly downregulated by eckmaxol treatment (Figure 5d–f).

### 2.7. Eckmaxol Inhibited the Nuclear Factor-κB (NF-κB) Nuclear Translocation and Mitogen-activated Protein Kinase (MAPK) Phosphorylation Induced via PM

**Figure 6:**
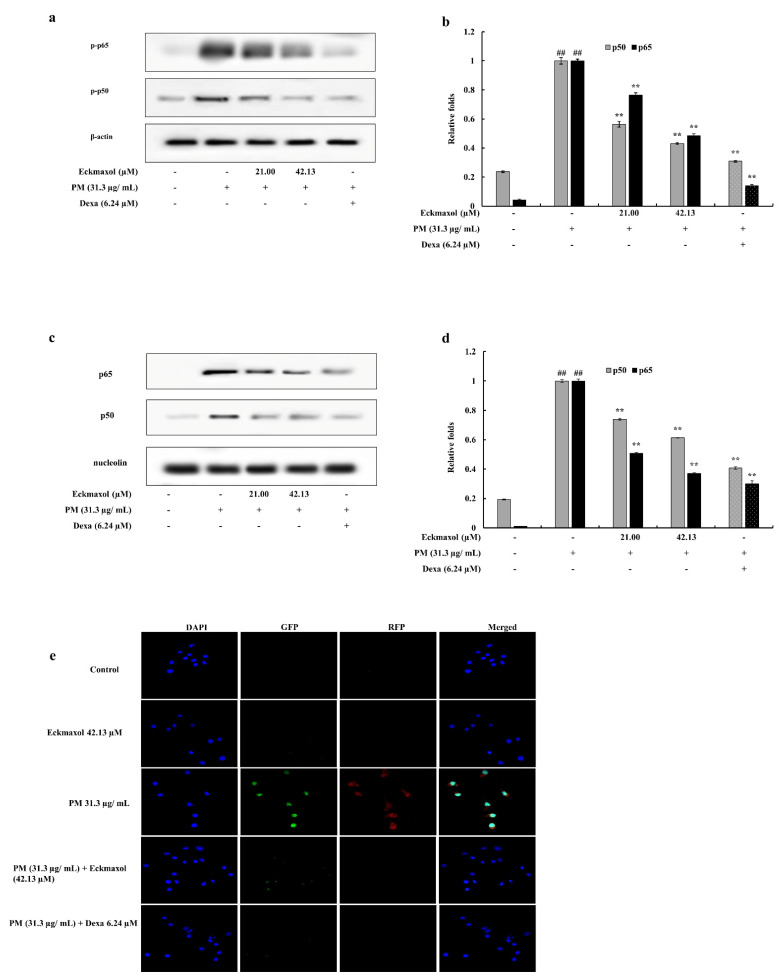
Influence of eckmaxol on the nuclear translocation of NF-κB in particulate matter (PM)-indued MH-S lung macrophages. (**a**) Phosphorylation of p65 and p50 in cytoplasm, (**b**) quantification of p50 and p65 in cytoplasm, (**c**) protein expression of p65 and p50 in the nucleus, and (**d**) quantification of protein expression of p65 and p50. (**e**) The cells were stained using 4′,6-diamidino-2-phenylindole (DAPI), green flourecesence protein (GFP), and red fluorescensce protein (RFP) stainings. Triplicate experiments were used to evaluate the data and the mean value is expressed with ± SD. ** *p* < 0.01, against PM-treated group or ## *p* < 0.01, against control (ANOVA, Duncan’s multiple range test). β-actin (cytoplasm) and nucleolin (nucleus) were used as an internal control. Quantitative data were analyzed using Image J software.

Nuclear translocation of NF-κB was evaluated by measuring the phosphorylation levels of NF-κB subunits p65 and p50 in the cytoplasm and their expression levels in the nucleus. As shown in Figure 6, phosphorylation of p65 and p50 in the cytosol was significantly increased by PM treatment. This upregulation was significantly decreased by eckmaxol treatment. The expression levels of these subunits in the nucleus were also significantly increased by PM exposure and downregulated by eckmaxol in a significant and dose-dependent manner. This was further analyzed using an immunofluorescence assay. Phosphorylation levels of p50 and p65 were detected using green and red fluorescence-conjugated secondary antibodies, respectively. According to the results, PM significantly stimulated the phosphorylation of p50 and p65 in the macrophages. However, eckmaxol significantly reduced the phosphorylation levels of p50 and p65. These results further confirmed the results of the Western blot analysis (Figure 6e).

**Figure 7:**
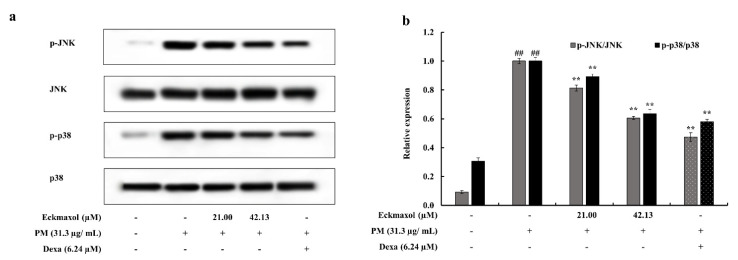
Evaluation of the effect of eckmaxol treatments on mitogen-activated protein kinase (MAPK) pathway proteins in pariculate matter (PM)-stimulated MH-S lung macrophages. (**a**) Western blot results of JNK, p38, and their phosphorylated forms and (**b**) quantitative data. Triplicated experiments were used to evaluate the data and the mean value is expressed with ± SD. ** *p* < 0.01, against PM-treated group or ## *p* < 0.01, against control (ANOVA, Duncan’s multiple range test). β-actin was used as a internal control. Quantitative data were analyzed using Image J software.

Phosphorylation of transcription factors, such as c-Jun N-terminal Kinase (JNK) and p38, leads to gene expression and cytokine production. PM significantly upregulated the phosphorylation of JNK and p38. However, this was significantly reduced by eckmaxol treatment. Thus, eckmaxol significantly downregulated PM-stimulated MAPK phosphorylation (Figure 7).

## 3. Discussion

The investigation of marine resources has become a major topic in the current scientific world. Marine algae are considered a major resource that consists of various secondary metabolites, such as polyphenols, polysaccharides, proteins, and peptides, which have many valuable bioactivities [29,30,31]. Among these marine algae, *E. maxima* contain various phloroglucinol-derived polyphenols such as eckmaxol, which exhibits valuable bioactivities, such as neuroprotective effects [18]. The results of the present study reveal the potential of eckmaxol as an anti-inflammatory agent against PM-induced inflammation in lung macrophages.

Dose-range determination analysis revealed a safe range of eckmaxol in MH-S lung macrophages. Furthermore, these optimized doses of eckmaxol exhibited cytoprotectivity by downregulating cell death and NO production in PM-induced MH-S lung macrophages. NO and PGE-2 act as inflammatory mediators and play a crucial role in chronic inflammation and host defenses. NO is derived from L-arginine through the enzymatic activity of iNOS and PGE-2 is generated by the enzymatic activity of COX-2 that converts arachidonic acid into PGE-2. Certain types of inflammation, such as asthma, generate NO at high levels, which acts as a pro-inflammatory agent, and COX-2 contributes to the production of autoregulatory, homeostatic prostanoid, and prostanoid release during inflammation. Therefore, regulation of NO and PGE-2 production is a versatile way to regulate inflammatory responses in macrophages [32,33]. The Western blot and qPCR results of iNOS and COX-2 solidified the anti-inflammatory potential of eckmaxol. Further, this solidified the potential of eckmaxol to down-regulate inflammatory mediators such as NO and PGE-2.

Small secreted proteins, called cytokines, are considered key regulators of inflammation. Cytokines are generated in response to invading pathogens and stimulate, proliferate, and recruit immune cells. Therefore, regulating cytokine levels could lead to manipulating the ultimate PGE-2 and NO production. The present study evaluated the regulation of PM-stimulated TNF-α, IL-6, and IL-1 production by eckmaxol based on their crucial role in inflammation [34,35]. ELISA results showed a significant up-regulation of these pro-inflammatory cytokine productions, significantly and dose-dependently declined by eckmaxol treatments. These results were consistent with the gene expression results of IL-1β, IL-6, and TNF-α. This further confirmed the anti-inflammatory activity of eckmaxol in the MH-S lung macrophages. These results emphasize the studying of the regulation of inflammatory signaling pathways by eckmaxol.

Therefore, the present study evaluated the activation of TLRs on the cell surface and endosomes to measure the effect of eckmaxol on inflammatory responses. The downstream signaling pathways initiated by TLRs activate the NF-κB and MAPK signaling pathways through myeloid differentiation primary response 88 (MYD88) and TNF receptor-associated factor 6 (TRAF6) [36]. Thus, identification of TLR activation and expression levels provides insight into PM-stimulated inflammation and the anti-inflammatory potential of eckmaxol. According to a previous study, TLR-2 and TLR-4 null mice stimulated by PM expressed lower levels of inflammatory responses than normal mice [37]. Furthermore, upregulation of TLR-7 by PM in MH-S lung macrophages has been previously reported [7]. This indicates the importance of TLRs in PM-stimulated inflammation. Gene expression evaluation in the present study highlighted that the upregulation of TLR-2, TLR-4, and TLR-7 by PM was significantly and dose-dependently decreased by eckmaxol treatment.

The uncontrolled production of pro-inflammatory cytokines and activation of cell signaling pathways, such as NF-κB and MAPK, play a pivotal role in pro-inflammatory responses [4]. NF-κB comprises a family of transcription factors that initiate gene expression to produce pro-inflammatory cytokines. Under non-stimulated conditions, NF-κB proteins such as p50 and p65 are bound to an inhibitor called nuclear factor of kappa light polypeptide gene enhancer in B-cells inhibitor, alpha (IκBα). This maintains p50 and p65 in their inactive forms in the cytoplasm. However, when a cell is exposed to the stimulators of pro-inflammatory mediators such as iNOS, COX-2, and pro-inflammatory cytokines, it initiates the translocation of these transcription factors from the cytoplasm to the nucleus and the transcription of genes responsible for pro-inflammatory cytokine production [38]. The current study evaluated the nuclear translocation of p50 and p65 following PM stimulation and the potential of eckmaxol for its regulation. Western blotting and immunostaining revealed that eckmaxol successfully downregulated the phosphorylation and translocation of p50 and p65. This finding strengthens the potential of eckmaxol as an anti-inflammatory agent.

The MAPK signaling pathway consists of numerous serine–threonine protein kinases that transfer signals from the cell surface to the nucleus to initiate gene expression, differentiation, mitosis, apoptosis, and survival [39]. Many studies have confirmed the important role of MAPKs, such as p38 and JNK, in inflammation-related gene expression [40,41]. Overall, regulating MAPK phosphorylation is a feasible approach to manipulate pro-inflammation and treat pro-inflammatory diseases. The phosphorylation of p38 and JNK was evaluated in the present study, which revealed that eckmaxol significantly decreased the PM-induced phosphorylation of these MAPKs. This suggests the potential of eckmaxol to regulate pro-inflammatory gene expression and cytokine production.

The experimental evidence provides mechanistic insight into the effect of eckmaxol against PM-induced pro-inflammation through evading extensive NO and PGE-2 production. Moreover, the involvement of inflammatory signaling pathways and cytokine production with PM exposure, and the effect of eckmaxol to attenuate them in MH-S lung macrophages, were confirmed. The findings further emphasize the potential of eckmaxol to develop an anti-inflammatory agent.

## 4. Materials and Methods

### 4.1. Chemicals and Regents

CRM-certified Chinese fine dust PM (CRM No.28 Urban Aerosols) was purchased from the Center for Environmental Measurement and Analysis, National Institute for Environmental Studies (Ibaraki, Japan). The murine MH-S lung macrophage cell line was purchased from American Type Culture Collection (Rockville, MD, USA). Roswell Park Memorial Institute medium (RPMI) supplemented with fetal bovine serum (FBS) and antibiotics (penicillin and streptomycin) were purchased from Gibco (Life Technologies, Grand Island, NY, USA). The antibodies used for Western blotting were purchased from Santa Cruz Biotechnology (Santa Cruz, CA, USA). The cytokine assay kits used for the experiment were purchased from eBioscience (San Diego, CA, USA), R&D Systems (Minneapolis, MN, USA), BD Optics (San Diego, CA, USA), and Invitrogen (Carlsbad, CA, USA). Unless otherwise noted, all chemicals were purchased from Sigma–Aldrich (St. Louis, MO, USA). HPLC-grade methanol and acetonitrile were purchased from Honeywell Burdick and Jackson, respectively (Muskegon, MI, USA). Analytical grade formic acid was obtained from Fluka Chemical (Buchs, Switzerland), and distilled water was purified from the Milli Q system (Millipore, Milford, MA, USA) used in this study.

### 4.2. Isolation and Characterization of Eckmaxol

Eckmaxol was isolated and purified as previously described with slight modifications. Briefly, *E. maxima* ethyl acetate fraction was used to isolate eckmaxol using centrifugal partition chromatography (CPC 240, Tokyo, Japan) with a ratio of n-hexane: ethyl acetate: methanol: water (3:7:4:6 *v*/*v*). The two phases were separated after the mixture had been thoroughly equilibrated in a separating funnel at room temperature (25 °C). The upper organic phase was used as the stationary phase and the lower aqueous phase was used as the mobile phase. The organic stationary phase was filled with the CPC phase and rotated at a speed of 1000 rpm. Subsequently, the aqueous mobile phase was pumped into the column in descending mode at a flow rate of 2 mL/min. Hydrodynamic equilibrium was maintained before sample injection, and 500 mg of *E. maxima* ethyl acetate fraction was dissolved in 6 mL 1:1 *v*/*v*. water: methanol and injected through the injection valve. An automatic fraction collector was used to collect the fractions (6 mL per tube) in the UV detection range of 230 nm. An HPLC system equipped with a PDA detector was used for further purification. A YMC-Pack ODS-A 10 × 250 mm, 5 μm column with acetonitrile + 0.1% formic acid and deionized water + 0.1% formic acid was used as the mobile phase at a flow rate of 2 mL/min [18,27,42].

### 4.3. Morphological Analysis of PM

First, the sample was coated with a platinum sputter (Quorum Technologies, Lewes, UK), and the surface morphology of the CRM No. 28 particles was observed using a JSM-6700F field-emission scanning electron microscope (JEOL, Tokyo, Japan). The device was operated at 10.0 kV [43].

### 4.4. Cell Culture

#### 4.4.1. MH-S Lung Macrophage Cell Culture

Murine MH-S lung macrophages were maintained in RPMI growth medium supplemented with 10% FBS and 1% antibiotics. Cells were maintained under controlled conditions of 5% CO_2_ at 37 °C. Cells were periodically sub-cultured and used in the exponential growth phase for the experiments [7].

#### 4.4.2. Cell Viability Assay and Dose-Range Determination for PM

The cytotoxic effects of PM, eckmaxol, and their combinations on MH-S lung macrophages were assessed using a colorimetric MTT assay. The experiment was performed according to the procedure described by Sanjeewa et al. (2020). MH-S lung macrophages were seeded at a concentration of 1 × 105 cells/mL in a 96 well plate. Eckmaxol (15.6–250 μg/mL) was treated after a 24 h incubation period. The cells were then treated for 1 h with PM (31.3 μg/mL) and incubated for 24 h again. The MTT assay was conducted to assess cell viability. Absorbance was measured at 540 nm using a Model 680 plate reader (Biotek Instruments, Inc., Winooski, VT, USA) [7,44].

#### 4.4.3. Determination of Nitric Oxide (NO) Production

A Griess assay was performed to evaluate the ability of eckmaxol to inhibit NO production in PM-induced MH-S lung macrophages. In brief, MS-H cells were seeded at a concentration of 1 × 10^5^ cells/mL in a 96-well plate, and eckmaxol was added after a 24 h period incubation period. After 1 h, PM was added, and incubation was continued for another 24 h under controlled conditions of 5% CO_2_ at 37 °C. An equal amount of Griess reagent was added to the culture supernatant and mixed in a 96 well plate. After 10 min of incubation, absorbance was measured at 540 nm [7].

#### 4.4.4. Evaluation of Pro-Inflammatory Cytokines and Prostaglandin E-2 (PGE-2) Production

MH-S lung macrophages were seeded and treated with different eckmaxol concentrations. After 1 h of incubation, cells were stimulated with PM for 24 h. The supernatant was collected to analyze pro-inflammatory cytokine levels, including cytokines (IL-1β, IL-6, and TNF-α) and PGE-2 production using ELISA kits [45].

### 4.5. Western Blotting

MH-S lung macrophages were seeded in a six-well plate and treated with different concentrations of eckmaxol after 24 h of seeding. The cells were then stimulated with PM for 1 h and incubated for another 24 h to collect the cells. The harvested MH-S lung macrophages were washed with ice-cold PBS, and cytosolic proteins were collected using a cytoplasmic and nuclear protein extraction kit (Thermo Scientific, Rockford, IL, USA) according to a previously described method [46]. After extraction, the protein content of each supernatant was determined using the BCA protein assay kit. Cellular proteins were separated by electrophoresis on 12% SDS-polyacrylamide gels and transferred to polyvinylidene fluoride (PVDF) membranes (GE Healthcare, Uppsala, Sweden). Membranes were blocked with 5% skim milk in TBST at room temperature for 2 h and incubated with primary antibodies in a cold room for approximately 8 h. Anti-inducible nitric oxide synthase (iNOS) and COX-2 were used for this experiment. p38, p-p38, p50, p-p50, p65, p-p65, JNK, p-JNK, nucleolin, and β-actin (1:1000) were used. After 8 h of incubation, the blots were washed twice with Ttween 20/Tris-buffered saline and incubated with the secondary antibodies for 45 min (1:3000). The bands were visualized using the FUSION Solo Vilber Lourmat system and band intensity was quantified using the ImageJ program.

### 4.6. Evaluation of the NF-κB Nuclear Localization

MH-S lung macrophages were seeded in Nunc^®^ Lab-Tek^®^ 8-well Chamber Slide™ (Nunc, NY, USA), treated with eckmaxol at concentrations of 15.6, 31.3, 62.5 μg/mL and stimulated with PM. MH-S lung macrophages were fixed with 4% paraformaldehyde for 5 min at room temperature and washed thrice with ice-cold PBST for 5 min each. The cells were then permeabilized with 0.1% Triton-X-100 and rinsed thrice with ice-cold water with PBST for 5 min. The cells were blocked with 10% donkey serum (Abcam, Cambridge, MA, USA) and incubated overnight with NF-κB p50 and p65 at 4 °C (1:200 in donkey serum). Next, fluorescent dye-conjugated secondary antibodies (Alexa Fluor^®^ 647, Abcam, Cambridge, MA, USA) were added and incubated for 2 h at room temperature, followed by three washes with ice-cold PBST for 5 min each. The prepared samples were incubated with 4’,6-diamidino-2-phenylindole (DAPI) nuclear stain (300 nM) for 10 min and washed thrice with PBST for 5 min to remove excess DAPI. Then, the coverslips were placed on chamber glass slides with Fluor Shield^TM^ histology mounting medium (Sigma-Aldrich, St. Louis, MO, USA). Images of the stained slides were captured using a Lionheart^TM^ FX Automated Microscope System (Bio-Tek Instruments, Inc., Winooski, VT, USA) [7].

### 4.7. Gene Expression Analysis

#### 4.7.1. RNA Extraction and cDNA Synthesis

Total RNA from MH-S lung macrophages was extracted according to the manufacturer’s instructions using TRIzol reagent (Life Technologies, Carlsbad, CA, USA). The total amount of RNA (1 μg) was reverse-transcribed using a first-strand cDNA synthesis kit (TaKaRa, Shiga, Japan) to obtain cDNA according to the manufacturer’s instructions. The cDNA was amplified using the primers listed in Table 1 (Bioneer, Seoul, South Korea) [47].

#### 4.7.2. Real-Time Reverse Transcription-Polymerase Chain Reaction (RT-PCR)

The conditions for the PCR amplification were as follows: one cycle at 95 °C for 10 s, followed by 45 cycles at 95 °C for 5 s, 55 °C for 10 s, and 72 °C for 20 s; and a final single cycle at 95 °C for 15 s, 55 °C for 30 s, and 95 °C for 15 s. The relative levels of target genes were calculated and normalized to GAPDH levels. All experiments were performed in triplicate. mRNA expression levels were calculated using the Livak method (2^−ΔΔCT^) (Livak and Schmittgen, 2001).

## 5. Conclusions

The results of this study provide important evidence of the effects of PM on inflammatory responses in lung macrophages. Here, the stimulation of inflammation in lung macrophages by PM and downstream activation through TLRs, NF-κB, and MAPK was demonstrated. Furthermore, the study confirmed that eckmaxol isolated from *E. maxima* significantly and dose-dependently inhibited PM-stimulated inflammation in lung macrophages via these receptors and signaling pathways. Taken together, the results of gene expression analysis and protein production provide clear insight into the potential of eckmaxol as an anti-inflammatory agent. In addition, further studies, including in vivo experiments and human trials, are required to confirm the use of eckmaxol as an anti-inflammatory agent against PM-induced pro-inflammation in the lungs.

## Figures and Tables

**Table 1:** Sequences of primers used in the present study.

Gene	Primer	Sequence
GAPDH	Sense	5′-AAGGGTCATCATCTCTGCCC-3′
	Antisense	5′-GTGATGGCATGGACTGTGGT-3′
iNOS	Sense	5′-ATGTCCGAAGCAAACATCAC-3′
	Antisense	5′-TAATGTCCAGGAAGTAGGTG-3′
COX-2	Sense	5′-CAGCAAATCCTTGCTGTTCC-3′
	Antisense	5′-TGGGCAAAGAATGCAAACATC-3′
IL-6	Sense	5′-GTACTCCAGAAGACCAGAGG-3′
	Antisense	5′-TGCTGGTGACAACCACGGCC-3′
IL-1β	Sense	5′-CAGGATGAGGACATGAGCACC-3′
	Antisense	5′-CTCTGCAGACTCAAACTCCAC-3′
TNF-α	Sense	5′-TTGACCTCAGCGCTGAGTTG-3′
	Antisense	5′-CCTGTAGCCCACGTCGTAGC-3′
TLR-2	Sense	5′-CAGCTGGAGAACTCTGACCC-3′
	Antisense	5′-CAAAGAGCCTGAAGTGGGAG-3′
TLR-4	Sense	5′-CAACATCATCCAGGAAGGC-3
	Antisense	5′-GAAGGCGATACAATTCCACC-3′
TLR-7	Sense	5′-TTCCTTCCGTAGGCTGAACC-3′
	Antisense	5′-GTAAGCTGGATGGCAGATCC-3′

## Data Availability

Not applicable.

## Supplementary Materials

The following supporting information can be downloaded at: https://www.mdpi.com/article/10.3390/md20120766/s1, Figure S1: Chromatogram of Liquid Chromatography Mass Spectrometry (LC/MS) analysis.
