# Supplementary material for: Eckmaxol Isolated from Ecklonia maxima Attenuates Particulate-Matter-Induced Inflammation in MH-S Lung Macrophage
Source: Mar Drugs. 2022 Dec 7;20(12):766. doi: 10.3390/md20120766 (PMC9785775; doi:10.3390/md20120766)
Supplement: Supplementary file 1 [file marinedrugs-20-00766-s001.zip › marinedrugs-2044330-supplementary.pdf]

a

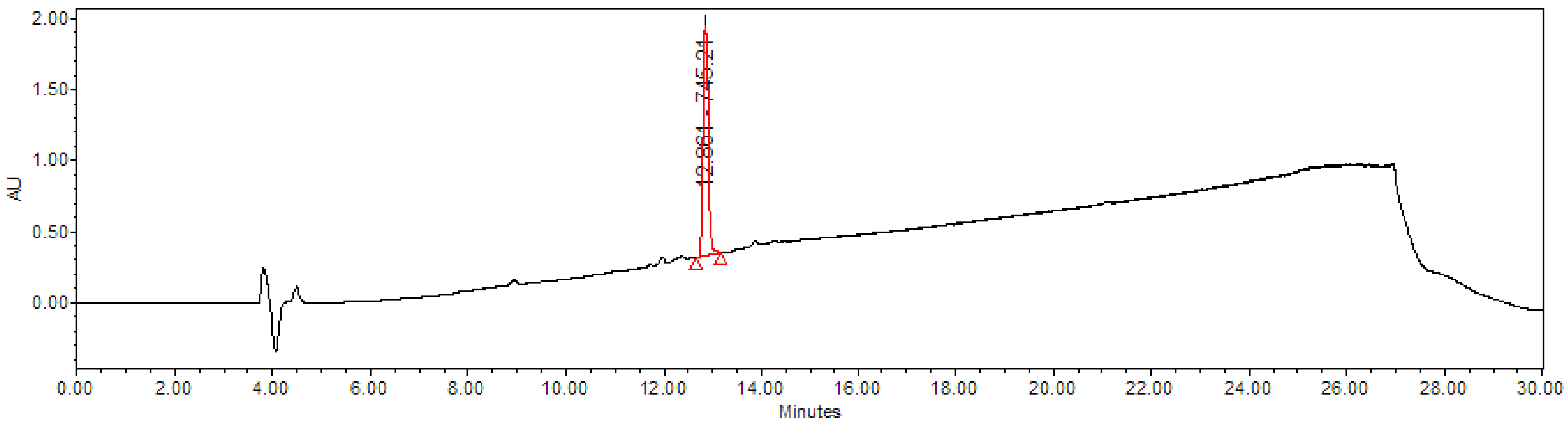

b

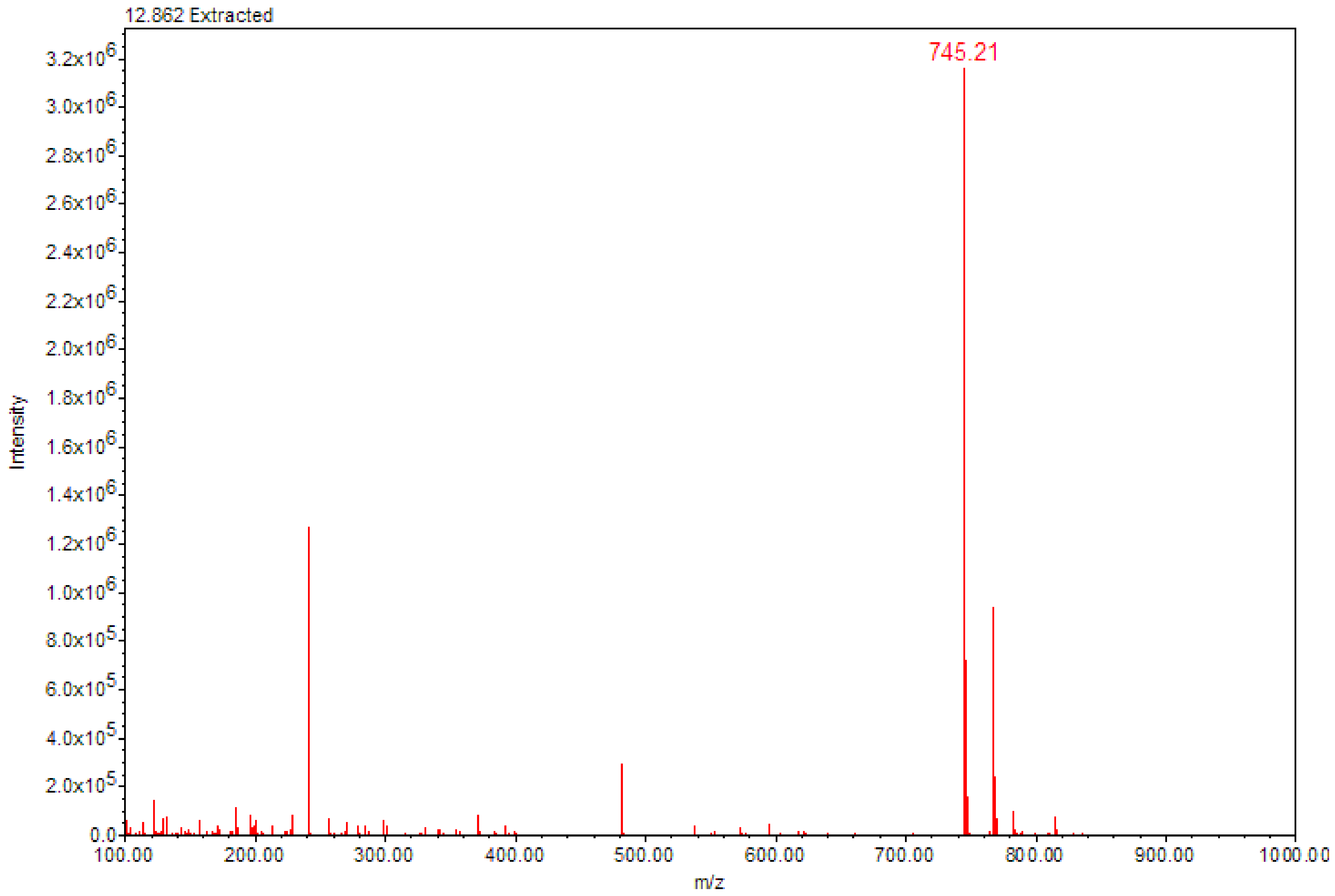

Supplementary Figure S1. Chromatogram of Liquid Chromatography–Mass Spectrometry (LC/MS) analysis , (a) HPLC chromatogram and (b) MS spectrum (ESI+)
